# Supplementary material for: Emulgel with Origanum vulgare L. Oil: A New Therapeutic Proposal in Case of Dermal Bacterial Infections
Source: Pharmaceuticals (Basel). 2025 Nov 20;18(11):1768. doi: 10.3390/ph18111768 (PMC12655586; doi:10.3390/ph18111768)
Supplement: Supplementary file 1 [file pharmaceuticals-18-01768-s001.zip › pharmaceuticals-3964876-supplementary.pdf]

Table S1. Chemical composition of *Origanum Vulgare* L. oil obtained by Gas Chromatography–mass spectrometry analysis (GC-MS).

No.	Compound	RT (min)	Rel. Area (%)	Method
1.	Ethanol	1.670	1.71	GC-MS
2.	Ethyl acetate	2.228	6.45	
3.	1-Butanol	2.571	2.65	
4.	Isobutyl acetate	3.694	1.37	
5.	2-methyl-, butanoic acid methyl ester	3.748	0.17	
6.	Butyl acetate	4.217	1.50	
7.	Furfural	4.496	0.10	
8.	a-Phellandrene, dimer	5.949	0.25	
9.	3,6,6-trimethyl-2-norpinene	6.078	1.54	
10.	Bicyclo[2.2.1 Jheptane, 2,2-dimethyl-3-methylene-, (IS)	6.323	0.56	
11.	1-Octen-3-ol	6.714	0.69	
12.	Bicyclo[3.1.1Jheptane, 6,6-dimethyl-2 methylene-, (IS)	6.772	0.25	
13.	B-Myrcene	6.925	3.53	
14.	3-Octanol	6.972	0.19	
15.	a-Phellandren	7.183	0.66	
16.	(1S)-2,6,6-Trimethylbicyclo〡3.I.I Jhept -2 - ene	7.282	0.23	
17.	(+)-2-Carenes	7.377	2.74	
18.	o-cymene	7.513	5.52	
19.	D-limonene	7.574	1.12	
20.	1,3,7-Octatriene, 3,7-dimethyl-	7.823	0.24	
21.	2-Thujen	8.040	6.24	
22.	beta-cis-Terpineol	8.180	0.41	
23.	1,4(8)-diee-para-mentha	8.248	0.12	
24.	cis-Linaloloxide	8.510	0.63	
25.	Linalool	8.663	10.18	
26.	Hortineol	8.710	0.44	
27.	endo-Borneol	9.734	1.48	
28.	(R)-(-)-p-Menth-1-en-4-ol	9.890	2.02	
29.	4-tertbutyl-o-cresol	10.790	0.33	
30.	Carvone	11.098	0.42	
31.	**Thymol**	**11.438**	**2.59**	
32.	**Carvacrol**	**11.620**	**21.29**	

33.	2-Methyl-5-(propan-2-ylidene) cyclohexane-1,4-diol	11.863	0.53	
34.	Caryophyllene	13.356	1.94	
35.	B-Bisabolene	14.353	3.15	
36.	(-)-Spatulenol	15.298	0.22	
37.	Caryophyllene oxide	15.393	0.41	
38.	Epizonaren	15.989	0.03	
39.	Decalin, 1-methoxymethyl	19.016	0.03	
